# The transformative power of structural predictions with AI in plant science

**DOI:** 10.1111/tpj.70807

**Published:** 2026-03-26

**Authors:** Joy Chenqu Lyu, Renier A. L. Van der Hoorn

**Affiliations:** ^1^ The Plant Chemetics Laboratory, Department of Biology University of Oxford Oxford UK

**Keywords:** AlphaFold, plant science, protein complexes, protein structure, structure prediction

## Abstract

Since the introduction of various structural prediction programs, the emerging transformative power of these technologies in plant science is apparent. Not only programs like AlphaFold but also RoseTTAFold, Chai‐1 and Boltz suddenly enable plant scientists to predict structures with high confidence. This ability has facilitated the discovery of novel protein functions inspired by structural homology and provided novel insights into how proteins evolved from ancestral folds. Prediction of protein oligomers and their interactions with lipids was crucial for studying immune receptors that assemble into resistosomes, while prediction of peptide–protein interactions has enabled the engineering of broad‐range cell surface receptors. *In silico* screens for novel protein interactions identified novel autophagy receptors and inhibitors of immune hydrolases. More discoveries will soon follow with the development of new tools to predict and analyse structures. These and many other recent discoveries highlight the transformative power of structural predictions with artificial intelligence in plant science.

## Significance Statement

Artificial intelligence (AI)‐based structural prediction is transforming plant sciences. High‐confidence protein models have accelerated functional discovery, illuminated evolutionary origins, enabled analysis of oligomers and receptor–ligand interactions and uncovered novel protein–protein interactions, illustrating a profound impact on plant science.

## INTRODUCTION

The rapid advancement of highly accurate artificial intelligence (AI)‐based protein structure prediction has transformed plant science research. In the past, structural insights of plant proteins were largely constrained by the limited availability of experimentally resolved structures. For instance, despite over 200 000 experimentally determined protein structures on Protein Data Bank (Berman et al., 2000), only around 8–10% of them are of plant origin. The underrepresentation of reliable structural references poses a significant bottleneck in functional, mechanistic and translational studies in plant protein research. However, the recent emergence of AI structural prediction tools has substantially increased access to high‐confidence structure models, enabling researchers to identify structural homologies of distant proteins, interrogate protein complex and protein–ligand interfaces and derive functional hypothesis of large protein families.

Structural analysis offers similarity comparisons on a three‐dimensional level for sequence divergent proteins (Balaji & Srinivasan, 2007). Proteins sharing a common fold may perform similar biochemical functions despite extensive sequence divergence, giving rise to protein families or ‘orphan’ proteins of unrelated sequences that cannot be annotated by comparative genomics. Following the release of the AlphaFold suite, large‐scale structural databases have been made accessible, providing high‐quality predicted models across entire proteomes facilitating the characterisation of unannotated proteins. In plant research, where protein annotation has often relied on homology to microbial or animal counterparts, structural prediction has enabled the discovery of previously unknown protein families in plants based on structural similarity (Buck et al., 2025) and assignment of novel protein functions through structural divergence (Huang et al., 2023). Given the substantial proportion of plant proteins with unknown functions, structure‐guided approaches remain a powerful strategy for functional annotation (Hanson et al., 2009).

Another key area of application of structure prediction is its use as substitutes for experimental models when the structures are predicted with high confidence, and atomic resolution is not essential to inform mechanistic hypotheses. Protein structures provide a framework for analysing protein–protein interactions and guiding mutagenesis to modify binding interfaces or chemical properties. For example, AlphaFold‐Multimer has been used to identify interactors of proteins of interests across different systems, with predicted models subsequently informing experimentally testable hypotheses (Homma et al., 2023; Lim et al., 2023; Poitras et al., 2023). Three‐dimensional shapes provided by the predicted models also facilitate identification of key residues for rational protein engineering. This approach is particularly useful in synthetic biology to improve enzyme stabilities (Yang, Xu, et al., 2025) or alter substrate specificities (Xia et al., 2024) and in therapeutics to evaluate drug targets (Najar Najafi et al., 2025). In plant sciences, these applications have been particularly prominent in studies of plant–microbe interactions (Homma et al., 2023; Ibrahim et al., 2023) and devising host receptor specificities during disease (Li et al., 2025; Snoeck et al., 2024; Zhang et al., 2025)—both of which have historically been difficult to dissect experimentally.

**Figure 1:**
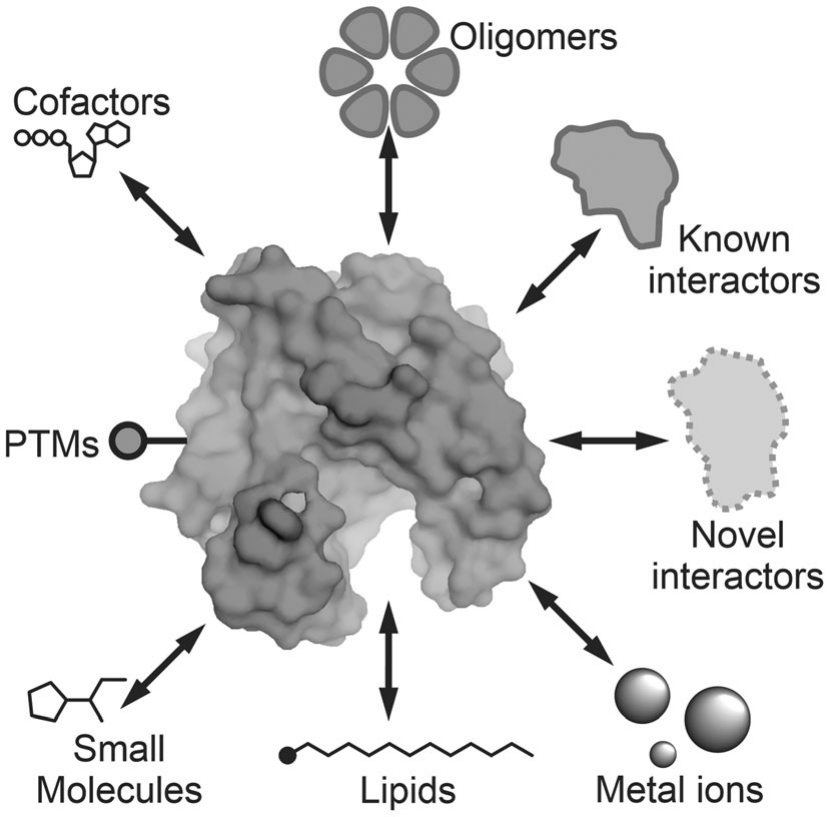
Eight types of artificial intelligence (AI)‐based structure predictions involving proteins. AI‐based predictions can not only predict the structure of a protein but also its interactions with itself (oligomers) or other proteins (heteromers) and interactions with cofactors, lipids, metal ions and other small molecules as well as post‐translational modifications (PTMs).

In this review, we highlight a few areas in plant science where AI‐predicted protein structures have accelerated discovery and facilitated hypothesis generation (Figure 1). We focus primarily on AlphaFold‐based predictions, given their early adoption and widespread use. Other AI‐based structural programs include Boltz‐1 (Wohlwend et al., 2025) and Chai‐1 (Chai et al., 2024). AlphaFold takes amino acids as inputs and generates predicted structural models with a few different quality metrics described in Box 1. We also discuss the usage of appropriate metrics in different cases.

Box 1 **Quality metrics of AlphaFold**

**Figure 2:**
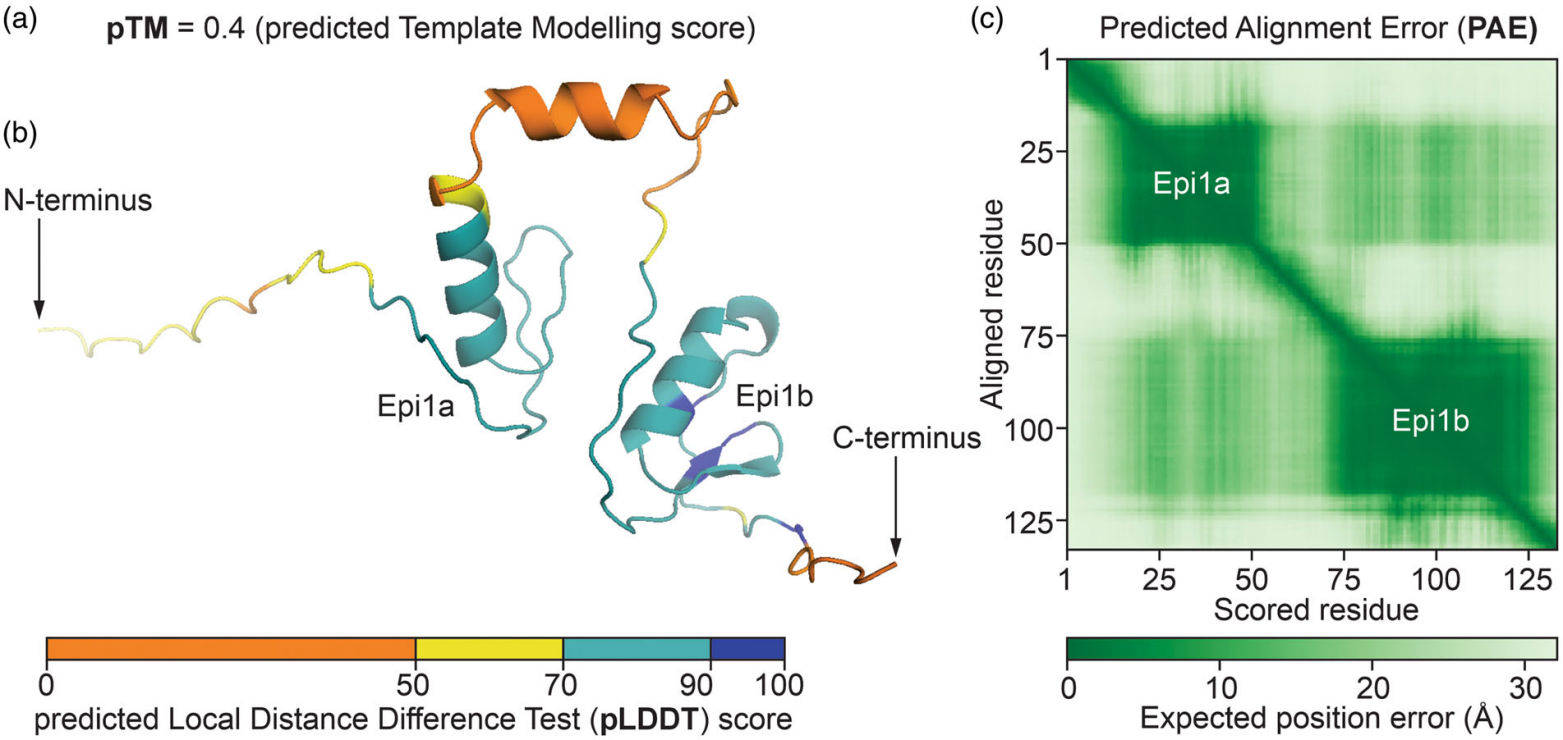
AlphaFold3‐predicted structure of Epi1 of *Phytophthora infestans* containing two Kazal‐like domains (Epi1a and Epi1b), illustrating the meaning of the pTM (a), pLDDT (b) and PAE scores (c).

So far, AlphaFold has been most frequently used in plant science. AlphaFold has four main types of output scores that we will use throughout this review, three of which are illustrated with a model of Epi1. The pTM is the *p*redicted *T*emplate *M*odelling score, which ranges from 0 to 1, indicating confidence of the general structure. The ipTM (also ranges from 0 to 1) summarises the confidence of all protein–protein interfaces of the structure. pTMs and ipTMs below 0.5 are widely considered as low confidence, whereas scores of 0.8 and above represent high confidence, although this can be context dependent. The pLDDT is the *p*redicted *L*ocal *D*istance *D*ifference *T*est, which is a per‐residue quality assessment ranging from 0 to 100, with higher pLDDT indicating greater confidence, and it is often represented in structures with different colours. The PAE is the *P*aired *A*lignment *E*rror, which represents the confidence in the relative position of two residues in the model, and is a useful indicator of domain packing. PAEs are expressed in angstrom (Å) and lower PAE indicates higher regional confidence. PAEs are often presented as a coloured 2D matrix summarising all the pairwise scores across all residues in the model (Figure 2).

## FUNCTIONAL ANNOTATION THROUGH STRUCTURAL SIMILARITY SEARCHES AND CLUSTERING

Accurate predicted structures provide valuable information enabling homology‐based searches on the structural level despite sparse sequence similarities. This is particularly helpful for developing hypothesis towards functional annotation of unknown sequences and identifying protein families that would otherwise be impossible to infer from sequence alignments.

**Figure 3:**
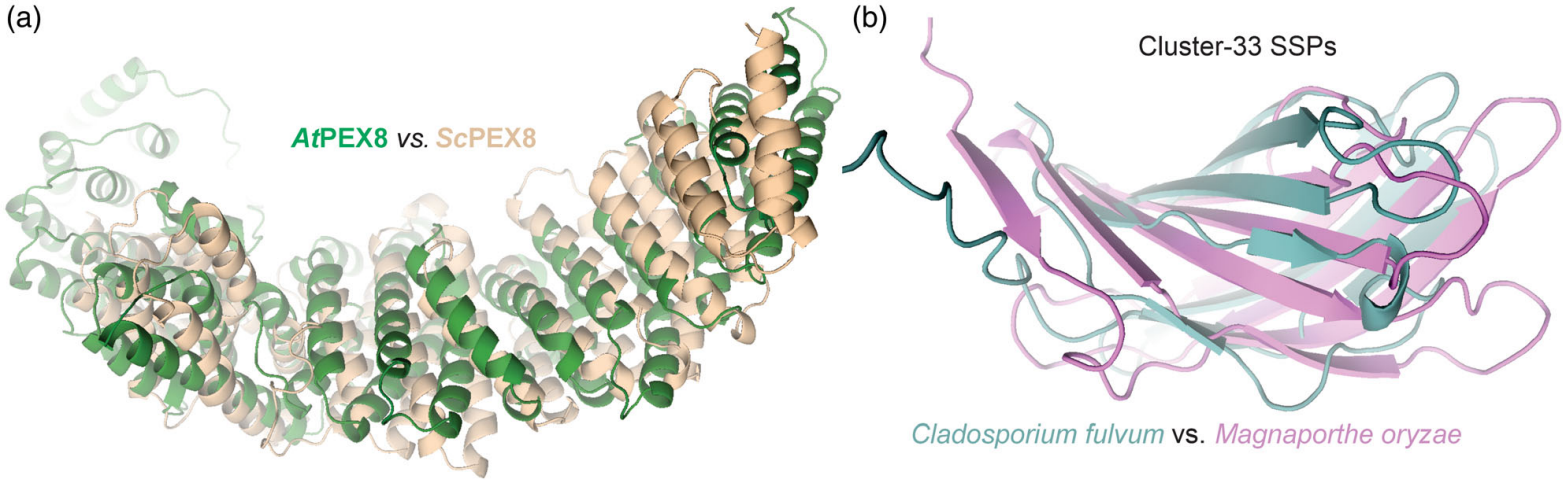
Using structural similarity clustering. (a) Peroxin PEX8 of Arabidopsis (*At*Pex8, green) was discovered upon a search for structures with highest similarity to yeast PEX8 (*Sc*PEX8, sand) even though they share only 11% reside identity. USalign structural comparison scores: RMSD = 4.95, TM: 0.72. (b) Two small secreted proteins (SSPs) secreted by different fungal pathogens share structural similarity. The Cluster‐33 proteins (Seong & Krasileva, 2023) are from tomato leaf mould pathogen *Cladosporium fulvum* (Clafu1_191139, green) and rice blight pathogen *Magnaporthe oryzae* (MGG_16836T0, purple) and share only 31% sequence identity. USalign structural comparison scores: RMSD = 3.61, TM: 0.58. Structures were predicted with AlphaFold3 (Table S1).

Buck et al. (2025), for instance, searched for structural homologues of yeast peroxisome import protein peroxin‐8 (*Sc*PEX8) in the AlphaFold Protein Structure Database (Varadi et al., 2022) with HHpred (Zimmermann et al., 2018), and identified Arabidopsis PEX8 (*At*PEX8), which was originally believed missing in plants. The *At*PEX8 has 100% probability of being structurally homologous and later experimentally proved to be functionally equivalent to *Sc*PEX8, but it shares only 11% sequence identity, making it otherwise impossible to be identified through sequence blasts (Figure 3a).

Another good example of structure‐guided functional annotation is that of Huang et al. (2023), who used AlphaFold‐predicted structures to cluster a family of 238 plant deaminases into subfamilies that correspond to their substrate preferences. This process identified a novel clade of deaminases that was found to target ssDNA but were originally annotated as dsDNA deaminases based on their sequences. This discovery greatly expanded the functional spectrum of plant DNA deaminases and laid the foundation of development of novel base editors in plants.

In addition to plants, two studies on plant–pathogenic fungi used structural similarity clustering to identify sequence‐unrelated but structurally similar (SUSS) families of small, secreted proteins (SSPs) and inferred evolution among distantly related sequences. Seong and Krasileva (2023) used AlphaFold2 to predict structures of 23 653 SSPs produced by 14 fungal pathogens and seven other microbes that they clustered with TM‐align (Zhang & Skolnick, 2005) and found evidence for effector evolution from ancestral folds that are conserved among fungi. Derbyshire and Raffaele (2023) also used AlphaFold2 to predict structures of 3925 SSPs for ascomycete plant pathogens and used DALI (Holm & Park, 2000) for similarity searches. Their analysis indicated that these SSP structures diversified through changes in patterns of thermodynamic frustration at surface residues.

These four studies illustrate the power of structural homology searches in the functional classification and annotation of proteins from plants and plant pathogens. One possible limitation from the above studies is that programs like DALI and TM‐align are relatively slow and therefore less scalable for proteome‐wide searches. A more recent development, FoldSeek (van Kempen et al., 2024), may represent a more practical option for structural clustering of large databases.

## NOVEL INSIGHTS INTO PATHOGEN EFFECTOR RECOGNITION

Many plants recognise invading pathogens through nucleotide‐binding leucine‐rich repeat (LRR) immune receptors (NLRs), which bind to effectors and initiate signalling cascades leading to immune responses. Identification of novel NLR–effector interaction often requires substantial forward genetics work, yet structural information from predicted models has delivered striking new insights in how effectors are recognised.

For instance, Sugihara et al. (2023) discovered *Magnaporthe oryzae* effector AVR‐Mgk1, which, despite its lack of sequence homology to known protein families, is perceived by Pik‐1, an NLR of the Pik‐family known to recognise a subset of MAX family effectors. Based on predicted structural models, AVR‐Mgk1 was found to adopt a fold characteristic of MAX family effectors and to interact with Pik‐1 through a conserved domain known to mediate MAX effector binding.

**Figure 4:**
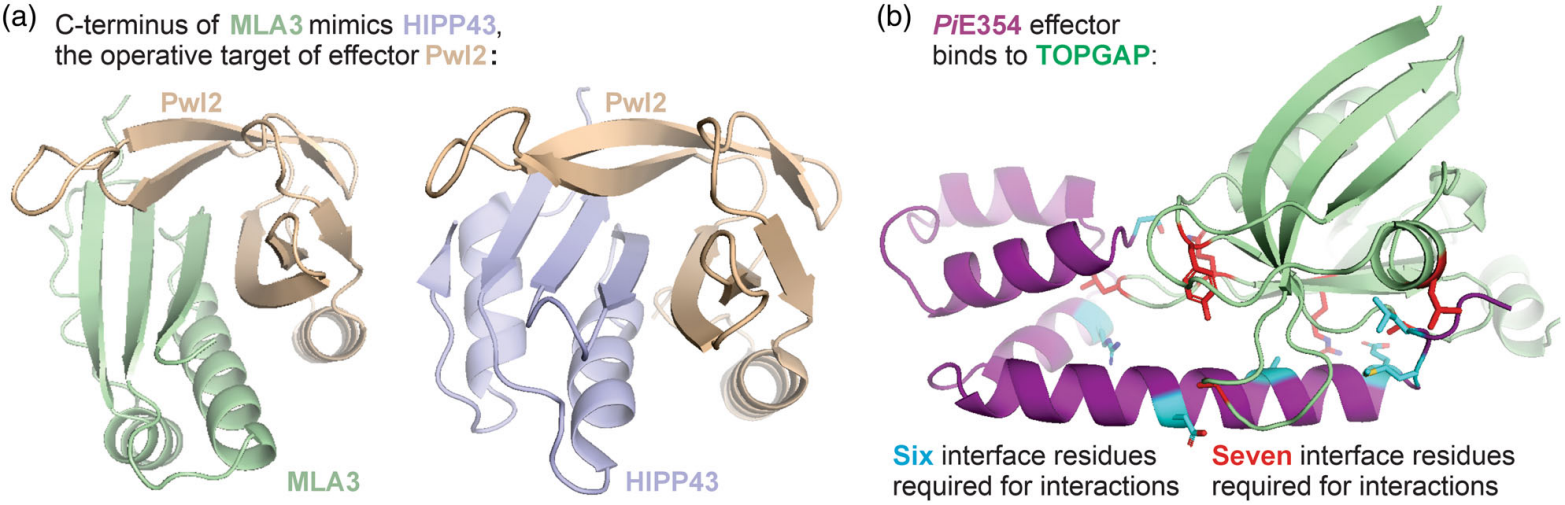
Novel insights into effector functions. (a) Immune receptor MLA3 mimics plant protein HIPP43, the operative target of effector Pwl2 (Gómez De La Cruz et al., 2024). The C terminus of immune receptor MLA3 of barley directly interacts with effector Pwl2 of *Magnaporthe oryzae*, with a structure that has striking similarity to how Pwl2 interacts with its operative target HIPP34 from barley, a heavy metal‐associated (HMA) domain. The Pwl2‐MLA3 structure was predicted with AlphaFold3 (Table S1), and the Pwl2‐HIPP43 was determined by crystallography (8R7A; Zdrzałek et al., 2024). (b) Effector *Pi*E354 promotes virulence by binding TOPGAP (Yuen et al., 2024). *Pi*E354 from the potato late blight pathogen *Phytophthora infestans* interacts with Rab GTPase‐activating protein TOPGAP of *Nicotiana benthamiana*. Substitution of six interface residues in *Pi*E354 into alanine abolished the interaction with TOPGAP and virulence function. Conversely, substitution of seven interface residues in TOPGAP into alanine abolished the interaction and *Pi*E354‐mediated virulence. This structure was predicted with AlphaFold3 (Table S1).

Gómez De La Cruz et al. (2024) discovered that plant immune receptor Mildew Locus A 3 (MLA3) confers the recognition of *M. oryzae* effector Pwl2 by mimicking its target in rice, HIPP42. Despite limited sequence homology, complexes predicted by AlphaFold2 revealed that the interface between HIPP42 and Pwl2 resembles that of the MLA3‐Pwl2 interface, revealing that MLA3 recognises Pwl2 by mimicking its virulence target, HIPP42 (Figure 4a).

Seong et al. (2025) used AI‐based structural models to restore recognition of an escaped wheat stem rust effector variant AvrSr50^QCMJC^ by a rye immune NLR Sr50. Through progressive optimisation of molecular docking models between Sr50 and AvrSr50, the authors identified three mutations in Sr50 that regained recognition to AvrSr50^QCMJC^. The authors noted that the structural model of AvrSr50^QCMJC^ with Sr50^Triple^ had a higher confidence score (ipTM) than that with the wild‐type Sr50, suggesting that AlphaFold may be able to distinguish gene‐for‐gene interactions in this case. Interestingly, Sr50 is also a close homologue of MLA3, which was discussed above. AlphaFold models indicated that the Sr50‐AvrSr50 interface is distinct from the MLA3‐Pwl2 interface, facilitating the construction of an Sr50 variant that can recognise both AvrSr50 and Pwl2 (Gómez De La Cruz et al., 2024).

These examples illustrate the power of AI‐driven structural predictions to elucidate perception mechanisms of effectors by NLR immune receptors.

## PREDICTING OLIGOMERISATION AND RESISTOSOMES

Protein oligomerisation is a widespread phenomenon where proteins assemble in higher order complexes consisting of multiple copies of themselves (homomers) or with other proteins (heteromers). Experimental methods to determine protein oligomeric states are tedious and time‐consuming. Interestingly, although the structure might not be fully accurate, AI‐based structural predictions are often better when the inputs have the correct subunit stoichiometry.

In a benchmarking study, AlphaFold2‐predicted PAE scores of interaction interfaces were used to identify homomers across proteomes from an archaeon, bacterium, yeast and human (Schweke et al., 2024). This study predicted that 20–45% of the proteins form homomers, and several of these predictions were confirmed experimentally. Likewise, Lin, Wallis, and Corry (2025) found that ipTM scores from both AlphaFold‐Multimer and AlphaFold3 are often the highest when proteins were predicted in their native oligomeric states. Together, these studies demonstrate that AI‐based structural prediction can be used to predict oligomers.

One particular use of oligomer protein prediction in plant science is for NLR immune receptors. Many NLRs act in pairs of sensors and helpers, which have specialised in pathogen detection and immune execution, respectively. When triggered, helper NLRs assemble into oligomeric complexes called ‘resistosomes’. Many resistosomes target membranes to trigger cell death, while others are assembled enzymes that generate signalling derivatives from nicotinamide adenine dinucleotide (NAD) (Abramson et al., 2024; Förderer et al., 2022; Liu et al., 2024; Madhuprakash et al., 2024; Wang et al., 2019; Zhao et al., 2022). The funnel‐shaped structure of the activated resistosome has been notoriously hard to resolve experimentally and was poorly predicted by AlphaFold2. This limitation has been significantly mitigated with the advancement of AlphaFold3. In studies of *Nicotiana benthamiana* (*Nb*) NLR *Nb*NRC2, AlphaFold2 frequently predicts the N‐terminal α helix of NLRs to be inward‐facing, whereas AlphaFold3 models it as an exposed tunnel. The residue contacts in the CC‐domain predicted by AlphaFold3 also largely agree with those observed in cryo‐EM structures (Madhuprakash et al., 2024). Although these predictions do still not fully recapitulate experimental accuracy, predicting structures of highly diverse CC domains enables comparative analysis in the absence of resolved structures, as in the example discussed next.

Canonical helper NLRs oligomerise into resistosomes and assemble CC domains with the N‐terminal MADA into a tunnel that punctuates the cell membrane and triggers cell death (Adachi et al., 2019). By contrast, for helper NLRs of the NRG (N requirement gene) clade, which lack the MADA sequence motifs, the mechanism that triggers cell death is not yet clear. Nevertheless, the predicted structure of a *N. benthamiana* NRG, *Nb*NRG1, revealed an extended N‐terminal ‘funnel’ that may be physically compatible with the membranes of other cellular compartments. This hypothesis was subsequently supported with subcellular localisation studies, which showed that *Nb*NRG1 forms puncta at chloroplasts, mitochondria and the endoplasmic reticulum, rather than at the plasma membrane (Ibrahim et al., 2024).

Interestingly, the increasing use of AI‐based structure predictions has led to the emergence of empirical, experience‐driven strategies for improving models and interpreting their outputs. When predicting NLR resistosome structures, for example, the model local confidence improves when 50 oleic acids are included as proxy for the membrane environment, and interactions are seen between the α1 helix and lipids (Ibrahim et al., 2024; Madhuprakash et al., 2024). Although the general applicability of this approach for membrane proteins remains unclear, adding ligands to approximate the local microenvironments might improve structural prediction of context‐dependent proteins. Consistent with above‐mentioned homomer predictions discussed above (Lin, Wallis, & Corry, 2025), the confidences for CC‐NLR resistosome models often improve when the input assembly matches their observed stoichiometry (Madhuprakash et al., 2024). Predicted resistosome structures for helper NLRs also often have higher confidence scores than those of counterpart sensor NLRs (Pai et al., 2025; Toghani et al., 2025). Although these predictions should not be taken as definitive structural evidence—for example, to determine resistosome stoichiometry or to distinguish helper and sensor NLR—they provide a convenient means of formulating hypotheses, prioritising experimental targets and informing functional annotation.

## IDENTIFICATION OF RECEPTOR–PEPTIDE INTERACTIONS

In addition to protein–protein interactions, AlphaFold has been used to predict ligands for plant surface receptors. This section focuses on pattern recognition receptors (PRRs), which perceive and respond to danger‐associated, pathogen‐associated or microbial‐associated molecular patterns (DAMP, PAMP or MAMP, respectively) from the environment (Ngou et al., 2022). PRRs typically comprise an extracellular domain responsible for ligand recognition, a transmembrane domain and a cytoplasmic domain that initiates downstream signalling. PRRs can be categorised into distinct families based on their extracellular and intracellular domains, with the largest PRR family employing horseshoe‐shaped LRR domains for ligand recognition (Albert et al., 2015). Although ligand recognition is often a dynamic process involving conformational changes that cannot be fully captured by static structural models, in *particular* cases, the predicted LRR–ligand complexes did provide insights to guide targeted engineering of receptor recognition.

For example, Snoeck et al. (2024) leveraged AlphaFold‐predicted models to delineate plant recognition of stress signals. In Arabidopsis, a group of 13–15 amino acid SERINE‐RICH ENDOGENOUS PEPTIDES (SCOOPs) is produced proteolytically from their precursors upon stress and is subsequently recognised by their receptor MDIS1‐INTERACTING RECEPTOR‐LIKE KINASE 2 (MIK2) (Hou et al., 2021; Rhodes et al., 2021; Zhang et al., 2022). To understand the recognition mechanism of diverse SCOOPs, Snoeck et al. used AlphaFold‐Multimer to model complexes of MIK2 with 50 Arabidopsis SCOOPs, from which they obtained 12 high confidence structures of ipTM >0.84. Across these models, SCOOPs were consistently predicted to interact with the same two binding pockets in MIK2 through their conserved SxS motif. In this case, AlphaFold3 generated complexes with higher scores, but the binding sites were similar to those predicted by AlphaFold‐Multimer. Subsequent experimental evidence supported these predictions because the degree of ligand perception was reduced when the binding pockets in MIK2 or SxS motifs were mutated.

The above study represents a nice application of structural prediction guided ligand identification; however, the applicability may be limited by the availability of resolved structures. The discussed case is strongly supported by resolved structures of MIK2‐SCOOP‐BAK1 complexes (Jia et al., 2024; Wu et al., 2024). For many other receptor–peptide interactions lacking resolved structural references, the confidence scores of the predictions are substantially lower (Li et al., 2025; Stevens et al., 2025). Nevertheless, as will be discussed in the next section, the ability to predict key interaction features highlights the potential of using predicted models as guides for engineering desired recognition.

## ENGINEERING PROTEIN–RECEPTOR INTERACTIONS

One of the key advantages of predicting interactions through structural models is the direct availability of interface information. Although they might not be completely accurate, they provide a useful starting point for functional characterisation through mutagenesis.

A recent study reported the discovery of a selective cold shock protein receptor (SCORE), which, as the name suggests, recognises the 15‐amino acid peptide (csp15) of conserved cold shock proteins (CSPs) (Ngou et al., 2025). Using AlphaFold models of csp15 peptides with SCORE and its co‐receptor BAK1, the authors identified several LRR motifs predicted to interact with csp15. Subsequent validation by domain swapping revealed that the 10th LRR motif primarily determines the recognition outcome. A close examination of protein model surface charges of different SCORE variants identified key charge variations on the ligand binding sites, particularly between the 8th and 10th LRR motifs, that correspond to charges in csp15 peptides. Building upon this observation, Ngou and colleagues generated a series of substitution variants with diverse surface charge properties and observed that the resulting ligand recognition profiles largely correlated with these engineered surface charges.

Similar studies were done with FLAGELLIN‐SENSITIVE 2 (FLS2), which recognises flg22, a 22‐residue peptide from bacterial flagellin (Gómez‐Gómez & Boller, 2000). A resolved structure of FLS2‐flg22 and the co‐receptor BAK1 is available (Sun et al., 2013), providing a good basis for structural prediction and engineering. Li et al. (2025) found that residues on the ligand‐binding surface on the LRR domains of FLS2 are highly polymorphic across different plant species, which have evolved to recognise diverse flg22 peptides from different pathogens. This implied high plasticity in the binding surface of FLS2 for precise engineering of recognition. Contact residues that were within 5 Å based on AlphaFold3 predicted models of FLS2, its co‐receptor BAK1/SERK3 and the corresponding flg22 epitope, were used to expand recognition of a narrow‐range receptor from *Fagus crenata Fc*FLS2 by substituting polymorphic interface residues from a broad‐range receptor from *Quercus variabilis Qv*FLS2.

In the two preceding sections, we discussed several well‐executed studies that used predicted receptor–ligand structures to generate key hypotheses. It is important to note that, while predicted models can be informative, they should not be taken as definitive evidence. This limitation is illustrated in another study of FLS2 published alongside the work discussed above, in which Zhang et al. (2025) reverse‐engineered FLS variant *Vr*FLS2^XL^ based on *Gm*FLS2b through domain swapping and site mutagenesis. They identified a minimal set of residues required to confer recognition of an evaded flg22 variant from *Gm*FLS2b into *Vr*FLS2^XL^. However, many of these residues did not map to the predicted interfaces between FLS2 and flg22 or BAK1 and were referred to as ‘dark matter polymorphisms’. These findings suggest that current structural predictions, however useful and convenient, may still miss certain aspects of receptor–ligand interactions, or that additional, unknown biochemical mechanisms also contribute to ligand recognition.

## DISCOVERY OF NOVEL PROTEIN–PROTEIN INTERACTIONS

Predicted structures can be a useful reference to identify candidates of novel protein–protein interactions, especially when they cannot be discovered though homologous sequence searches. This is particularly useful when the expected interface is too small to conduct proper sequence search, or the interacting proteins share limited homologies with other proteins. In this section, we review two independent studies that focused on interactions of very different protein types—autophagy receptor interactions and protease–inhibitor interactions—but were both built upon AlphaFold‐Multimer predictions.

**Figure 5:**
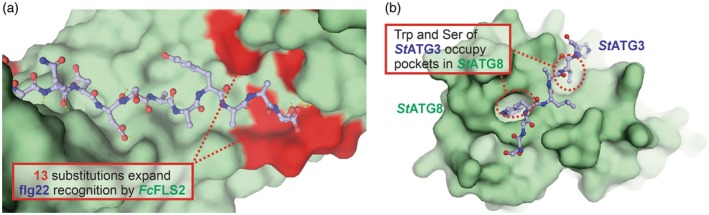
Predicting peptide–protein interactions. (a) Artificial intelligence (AI)‐guided receptor engineering expands bacterial recognition (Li et al., 2025). Cell surface receptor kinase FLS2 from Japanese beach, *Fagus crenata* (*Fc*FLS2, green surface model) was engineered with 13 substitutions from oak tree *Quercus variabilis* FLS2 (*Qv*FLS2, red residues), such that is recognised the 22‐residue fragment of flagellin of bacterial wilt pathogen *Ralstonia solanacearum* (flg22, ball and stick, only C‐terminal half is shown). This structure was predicted with AlphaFold3 (Table S1). (b) Peptide of *St*ATG3 docks into *St*ATG8. A β‐strand autophagy protein ATG3 of potato *Solanum tuberosum* (*St*ATG3, blue) carries two residues (Trp and Ser) that dock into two hydrophobic pockets in potato ATG8 (*St*ATG8, green) (Ibrahim et al., 2023). This structure was predicted with AlphaFold3 (Table S1).

Autophagy is a conserved eukaryotic process to degrade cellular materials and maintain homeostasis, and it is regulated through the autophagy‐related proteins (ATGs). ATG8 family proteins, for example, are heavily involved in specific interactions with cargo receptors, which enable subsequent formation of autophagosome and degradation on selected cargo types. Most ATG8 interacting proteins contain canonical, short, linear ATG8‐interacting motifs (AIMs) consisting of four amino acids, with the first aromatic and the last hydrophobic residue interacting with two hydrophobic pockets on ATG8. Identifying functional relevant AIMs though sequence search is almost impossible as there would simply be too many false positives. Ibrahim et al. (2023) leveraged structural predictions and identified both canonical and non‐canonical AIMs through occupancies of the two hydrophobic pockets on predicted structures. This approach also revealed an interaction between potato ATG3 and ATG8 through a noncanonical AIM motif that was confirmed experimentally (Figure 5b). This study highlighted that, for well‐modelled and understood interaction motifs, interaction predictions can be made at residue‐level resolution rather than solely at the level of overall protein–protein complexes. Accordingly, in such cases, per‐residue confidences (e.g. pLDDT of the interacting amino acids) would be key indicators of model relevance and should be more informative than global model quality metrics (e.g. pTM), especially when the interacting motifs are part of intrinsically disordered domains.

Another study of Homma et al. (2023) nicely demonstrated the use of large‐scale structure predictions to screen novel interactions during host–pathogen interaction. In the study, Homma and colleagues screened 1879 pathogen‐produced SSPs for interactions with six defence‐related hydrolases secreted by tomato, to identify candidate pathogen inhibitors of the host proteins. Manual selection over high confidence SSP–hydrolase complexes resulted in several unrelated SSPs that were predicted to occupy the active sites, and among them, four indeed suppressed labelling of immune protease P69B by active‐site targeting probes. The inhibitor candidates were diverse in sequences and structures and were predicted to target different surface residues on host proteins. This study highlights the power of structure prediction as a convenient, *in silico* approach to explore novel protein interactors, especially when sequence homology is sparse.

Collectively, these two studies demonstrated the potential of using predicted protein complex structures to identify novel interactors. In both cases, however, the authors focused on relevant interfaces, where high confidence likely indicates biologically relevant interactions. The predictability of computationally derived structural models on unknown interfaces is yet to be determined.

## ENABLING STRUCTURE‐GUIDED MUTAGENESIS

Beyond protein interaction prediction, structurally predicted interfaces can serve as valuable guides for targeted mutagenesis to disrupt interactions.

Yuen et al. (2024) found that AlphaFold accurately predicts the residues relevant for interaction between *Phytophthora infestans* effector *Pi*E354 and its host target TOPGAP, a Rab GTPase‐activating protein. To confirm the relevance of these interactions, they created non‐interacting *Pi*E354 by mutating six interface residues, which demonstrated that the interface is essential for promoting virulence. Likewise, they created a non‐interacting TOPGAP mutant by mutating seven interface residues for the reciprocal control (Figure 4b), confirming again that these residues are essential for binding *Pi*E354. Further AlphaFold3 modelling with GTPase Rab8a, the substrate of TOPGAP, suggested that *Pi*E354 alters the orientation of TOPGAP such that it can no longer interact with Rab8a, explaining how *Pi*E354 disrupts Rab8a function.

Xia et al. (2024) used AlphaFold Multimer models to engineer a soybean‐secreted inhibitor of pectin methylesterase (PME) called *Gm*PMI1. *Gm*PMI1 suppresses cell wall weakening by a *Phytophthora sojae* PME (*Ps*PME1), which is thought to protect host cells during infection. However, *Gm*PMI1 also suppresses the endogenous soybean *Gm*PME1; hence, the constitutive expression of *Gm*PMI1 to improve plant resistance will likely impose fitness costs. To avoid targeting of endogenous PME, Xia and colleagues compared AlphaFold Multimer models of *Gm*PMI1‐*Ps*PME1 and *Gm*PMI1‐*Gm*PME1 complexes, selected and mutated nine interface residues and created a *Gm*PMI1 variant that cannot inhibit *Gm*PME1. The amino acid residues chosen for mutagenesis were based on the ones from a *Nicotiana Gm*PMI1 homologue that does not inhibit PMEs.

Taking together, structural predictions can identify relevant interfaces and residues involved.

## FUTURE PERSPECTIVES

AI‐based structural predictions in plant science are only at their beginning, with much of its potential remaining to be explored. There are several structural features that can already be predicted that remain to be used and explored in plant science. Interactions with nucleic acids, for example, could help to predict targets of transcription factors (TFs). Yang, Tian, et al. (2025), for instance, characterised TFs of the *PLATZ* family in rice, using AlphaFold3‐predicted protein structures docked with A/T‐rich *PLATZ* binding sequences from pea. However, Lin, Huang, et al. (2025) noticed that AlphaFold3 was unable to accurately predict the DNA motif bound to TF ORE1 of Arabidopsis. More benchmarking studies are needed to establish standards for prediction accuracy of RNA/DNA–protein interactions.

Likewise, predicting interactions with metal ions is important to interpret protein functions. Metal ions were omitted from the training set for AlphaFold2 and RoseTTAFold, but binding sites for zinc and iron ions could at that time be predicted by the clustering of cysteine and other residues (Wehrspan et al., 2022; Willems et al., 2023). Binding sites for other metal ions by filling spaces are more challenging to predict. Although AlphaFold3 and RoseTTA‐AllAtom are trained on metal‐containing structures and are able to predict sites for metal ion binding, these programmes still require users to specify types and numbers of ions as inputs, and the generation of these models therefore requires prior knowledge of the studied proteins. Tools for explorative studies for binding metal ions remain to be developed to predict, for instance, the impact of particular ions on a plant proteome.

Furthermore, predicting interactions with small molecules is of great interest to both plant and medical science. In the past, molecular docking has been one of the most commonly used computational methods to predict protein interactions with other molecules. Molecular docking studies with AlphaFold2‐predicted structures were effective to predict therapeutic drug binding (Lyu et al., 2024). In plant science, docking with an AlphaFold‐predicted model of Arabidopsis O‐GlcNAc transferase SPINDLY (SPY) was used to identify an SPY inhibitor from a large virtual chemical library (Aizezi et al., 2023). The later released AlphaFold3, RoseTTAFold‐AllAtoms and Boltz can predict interactions between user‐provided small molecules and proteins. For example, Boltz‐2 was able to predict a high‐confidence structural model of plant β‐galactosidase BGAL1 in complex with iminosugar glycosyrin, a BGAL1 inhibitor produced by *Pseudomonas syringae* (Sanguankiattichai et al., 2025). The full potential of these tools for discovery of small molecule interactions in plant science has yet to be explored, for instance to predict targets of bioactive small molecules.

In addition, the latest AI‐based prediction algorithms can also predict post‐translational modifications including phosphorylation, glycosylation, acylation, methylation and nitrosylation. Sun et al. (2025) predicted phosphorylation sites on an Arabidopsis cyclic nucleotide‐gated channel component CNGC2, however, not through direct modelling of CNGC2 but the predicted interface with receptor kinase P2K1. AlphaFold3, RoseTTA‐AllAtom and Boltz can now handle user‐specified PTMs at specific residues, but some PTMs are not yet available, and the prediction of PTMs in a given protein or proteome remains a future challenge.

Besides some limitations listed above, there are two more areas where AI‐based structural predictions are limited. The prediction of structural changes caused by mutations is often not accurate. Even subtle mutations can lead to significant alteration in protein conformations, but such is not always reflected on the predicted structures. Lin, Huang, et al. (2025) and Lin, Wallis, and Corry (2025) highlighted an example of the Arabidopsis potassium channel SKOR D312N/L271P double mutant, whose predicted structure by AlphaFold3 aligns poorly to the published cryo‐EM structure. Nevertheless, AlphaFold3 predicted models correlated well with experimental data in binding‐free energy changes upon mutation in protein–protein complexes (Lu et al., 2024; Wee & Wei, 2024).

A second limitation is that conformational changes are often not predicted by AI‐based structural predictions. One discussed example is E3 ubiquitin ligase, which naturally adopts a ligand‐free open state and a ligand‐bound close state. AlphaFold3 was only able to capture the closed conformation (Abramson et al., 2024). In some cases, predicted structures complement experimental structures, as seen for Arabidopsis phosphate transporter *At*PHO1—experimental structural elucidation by cryo‐EM revealed a closed state, whereas AlphaFold predicted the open conformation (Fang et al., 2025). Some research labs have reported that subsampling of the multi‐sequence alignment during prediction can generate conformation‐specific output populations (Monteiro da Silva et al., 2024; Sala et al., 2023).

In conclusion, AI‐based structural predictions are a powerful new tool in the arsenal for the plant scientist and further developments will undoubtedly transform this research field even further.

## CONFLICT OF INTEREST

None declared.

## Supporting information


**Table S1.** Protein sequences used for AlphaFold3 predictions.

## Data Availability

No data were generated for this manuscript. Structural models for the figures were generated with AlphaFold3 using the sequences listed in Table S1.
