# Supplementary material for: The transformative power of structural predictions with AI in plant science
Source: Plant J. 2026 Mar 26;125(6):e70807. doi: 10.1111/tpj.70807 (PMC13020896; doi:10.1111/tpj.70807)
Supplement: Supplementary file 1 — Table S1. Protein sequences used for AlphaFold3 predictions. [file TPJ-125-0-s001.docx]

**Supplemental Table-I** Protein sequences used for AlphaFold3 predictions.

| AtPEX8 | MARKANNSFFLEEWLRTVSGSSVSGDLVKQNSAPSARSIIQAWSEIRESLQNQNFDSRYLQALRALVSSESTIHVADPQAKLLISILAFQDVSLPSESYTLVLRLLYVWIRKAFRPSQALVGVAVQAIRGVVDDRRNLQPALVAQSVLVSGAFACVPSLSGDVKVLCLELLCRLLEEEYSLVGSQEELVPVVLAGIGYALSSSLDVHYVRLLDLLFGIWLKDEGPRGTVTYGLMILHLIEWVVSGYMRSNSINKMSLFANEVLETSKEKYAVFAVFMAAAGVVRASTAGFSSGAQSLEISKLRNSAEKRIEFVAQILVSNGNVVTLPTTQREGPLLKCFAIALARCGSVSSSAPLLLCLTSALLTQVFPLGQIYESFCNAFGKEPIGPRLIWVREHLSDVLFKESGAISGAFCNQYSSASEENKYIVENMIWDFCQNLYLQHRQIAMLLCGIEDTLLGDIEKIAESSFLMVVVFALAVTKQWLKPIVSKERKMVTSVKILVSFSCVEYFRHIRLPEYMETIREVISCVQENDAPCVSFVESIPAYDSLTNPKDLFTQRIKYEWSRDDVQTSRILFYLRVIPTCIGRLSASAFRGVVASTMFLYIGHPNRKVAQASHTLLAAFLSSAKESEEDERTQFKEQLVFYYMQRSLEVYPEITPFEGLASGVATLVQHLPAGSPAIFYSVHSLVEKASTFSTESLQGRKSDPGNQILELLLRLVSLVDIQVLPYLMKSLAQLVIKLPKERQNVVLGELYGQVAESDDVIRKPSLVSWLQSLNYLCSNNRTEVLASGSTIDTSNQLAARL |
| --- | --- |
| ScPEX8 | MFDHDVEYLITALSSETRIQYDQRLLDEIAANVVYYVPRVKSPDTLYRLVGALFRSQFIVQLPPLRLLHIVKDVFLWKLEVSEPTLPISKFYLVWNAVFESHRATWNLSQLMVLDGVLVTYPSFKQLNNAYFIDESSNKTALYYRNWKLQLFSPIWAQLWNTAIVRANLSIQHCLLIALALLFNQSNRSALLHGVDVSWNLVTEKLLDLLEEYVHGIVQPMEIFSTDSVLSTNLNHLASCLTSSITRSNEATLVNSVRKLERICRYLSDTVASLKEQQLDFKFQNVFILIILALKELSAMNMTILPNHKDTFYSMICLSLFHVHVLTQKIGTVGFPSYDYVYDNLVTYFIVMDDLSKITTVLELMKRNNTKQDPNKLVFYINFLNKITNYYGCRIRLPFITEFIEPLLHFDVFFSGKTGNTLDIEIKESIHTLTITVLSIDSSYSSQVAQWQVSRILVYLKMSMDQFIAGKLSANQILLIFGHLSTQLPSLHNYNKHLLRDSLHETYIRIVNVKNPEKKNVLIECLIVQIAFINNPHHLIGWLNICLQLINTHNKKLLQQLWEMVSSLESSLAIDWWYTTVLSSQSSKL |
| MGG_16836T0 | APTAVSLLAAGPMDPTPVNVLMMKPGQFNYNWTVTNWEAGCARGGCYYGFDIAADEYTSYPKAPAFKAHCNGQNEGGPYTVCEMQDGGSAARRVAARLLERNSSDPVYIRVSYQYADSQPNAYFNWTGTGETSYNQFVAPPQTFNVTPSEVFGVA |
| >Clafu1_191139 | APAADPATTSMMAAGPPWTIQNFKRTCGGGVCTYDYAINQNNKATKCTYQVKGNPATRASYSNVKCGPYVISSNWSGQFGEGNGFQTLAVVNGKQIIYPAYTDKQLVNGQVVKPDQSYAPQNLP |
| MLA3 | LPTGEAVRFSLGVRVAKEDGNGGFDLGLQGNLLSLRRDYSVFIYCGGATVGEAKEAEAVVRRALKADPHVYYISIQMRPR |
| Pwl2 | GGGWTNKQFYNDKGEREGSISIRKGSEGDFNYGPSYPGGPDRMVRVHENNGNIRGMPPGYSLGPDHQEDKSDRQYYNRHGYHV |
| HIPP43 | RPLQTVNIKVKMDCEGCERRVKNAVKSMRGVTSVAVNPKQSRCTVTGYVEASKVLERVKSTGKAAEMW |
| PiE354 | VHPLLMHEGVAVAIAKSVSADLDSRLMAKVSQIDNGKEILKQWRDKGLTIAKLKPLLKNTKKWKATPERAVYNLLKKERFS |
| TOPGAP(RBD) | GVEIVYLKDNVAIHPTQHAWERIRGRLKLIKQGSSLLMTWIPYKGQSSSARLSEKDKSLYTIRAVPFSDIRSIRRHTPTLGWQYAIIVLLSGLAFPPLYFYNGGLKEFLATIKQHAFLVRSAEDANIFLVNDFQDPLQRTLSSLELPGAVSVANS |
| eFcFLS2 | SLEVQLEALKAFKNSITNDPSGALVDWTDRNHHCNWSGIACDPSSNYVVSISLIDKQLKGEITPFLGNLSSLQVLDLTSNTFTGHIPAQLGLCSQLSVLTLYENSLSGPIPPELGNLKNLQSVDLGSNFLNGSIPESICNCTSLLACSVSFNNLAGIIPSNIGNLANLQIFLVYKNNLVGSIPVSIGRLESLQALDLSQNQFSGVIPPQIGNFSNLEYLLLFENSFVGKIPSELGRCKKLVALELYTNQFTGGIPFEIGNLVHLETLRLYNNKLNSTIPASLLQLKSLTHLGLSENELTGIIPSELGSLRSLVVLTLHSNKFSGEIPSSLTNLTNLTQLSMSFNFLTGKLPSDIGLLYNLKNLTMNNNLLEGTIPSSIKNCTHLLVVGLSRNKITGKIPWGFQQLLNLTFLSLSYNKMSDEIPDDLFNCSNLRILDLTQNFFSGPIKSGIGKLSNLQILRVHTNSFIGPIPPDMGNLSKLFSLSLGQNSLSGLVPSELSKLSLLQGLYLNDNALEGAIPEKIFEVKQLNEVGLQNNRFKGPIPDAVSKLEQLSYLDLHGNMLNGSILRSMEHLNKLMTLDLSHNHLTGSIPSLVIASMKTMQIYLNLSYNFLVGTIPDELGTLETVQAIDISNNNLSGIIPKTLRGCRNLFSLDLSGNKLSGPIPAEALTQMDLLTSLNLSRNKLDSGLPEDLASLKHLISLDLSQNQLKGIIPESFANLSTLKHLNLSFNQLEGHVPEIGIFRHINSSSLMGNPDLCG |
| flg22(Rs) | QRLSTGLRVNSAQDDSAAYAAS |
| StATG3 | MVLSQLHGAFKDTVERITSPRTVSAFKEKGVLSVSEFVIAGDNLVSKCPTWSWESGESSKRKSYLPSDKQFLITRNVPCLRRAASIEEEYEAAGGEVLLDNEDNDGWLATHGKPKENNGAEDDNLPSIDASEINKKNTIQSIPSYFGGEEEEDIPDMGEYEEADNLIETDPATLQTTYLVANEPDDDNILRTRTYDVSITYDKYYQTPRVWLTGYDESRILLQPELVLEDVSQDHARKTVTIEDHPHLPGKHASVHPCRHGAVMKKIIDILMLRGVEPEVDKYLFLFLKFVASVIPTIEYDYTMDFDLGSSST |
| StATG8 | MAKSSFKLEHPLERRQAEAARIREKYPDRIPVIVEKAERSDIPDIDKKKYLVPADLTVGQFVYVVRKRIKLSAEKAIFIFVKNILPPTAAMMSAIYEEHKDEDGFLYMTYSGENTFGSF |
